# Complete Protection of Mice against Lethal Murine Cytomegalovirus Challenge by Immunization with DNA Vaccines Encoding Envelope Glycoprotein Complex III Antigens gH, gL and gO

**DOI:** 10.1371/journal.pone.0119964

**Published:** 2015-03-24

**Authors:** Huadong Wang, Chaoyang Huang, Jinrong Dong, Yanfeng Yao, Zhenyuan Xie, Xueying Liu, Wenjie Zhang, Fang Fang, Ze Chen

**Affiliations:** 1 College of Life Science, Hunan Normal University, Changsha, 410081, Hunan, China; 2 State Key Laboratory of Virology, Wuhan Institute of Virology, Chinese Academy of Sciences, Wuhan, 430071, Hubei, China; 3 Shanghai Institute of Biological Products, Shanghai, 200052, China; 4 Xie Tu Community Medical Service Center, Xuhui District of Shanghai, Shanghai, 200030, China; 5 Xinhua Hospital affiliated to Shanghai Jiaotong University of Medicine, Shanghai, 200092, China; Thomas Jefferson University, UNITED STATES

## Abstract

Human cytomegalovirus infects the majority of humanity which may lead to severe morbidity and mortality in newborns and immunocompromised adults. Humoral and cellular immunity are critical for controlling CMV infection. HCMV envelope glycoprotein complexes (gC I, II, III) represent major antigenic targets of antiviral immune responses. The gCIII complex is comprised of three glycoproteins, gH, gL, and gO. In the present study, DNA vaccines expressing the murine cytomegalovirus homologs of the gH, gL, and gO proteins were evaluated for protection against lethal MCMV infection in the mouse model. The results demonstrated that gH, gL, or gO single gene immunization could not yet offer good protection, whereas co-vaccination strategy apparently showed effects superior to separate immunization. Twice immunization with gH/gL/gO pDNAs could provide mice complete protection against lethal salivary gland-derived MCMV (SG-MCMV) challenge, while thrice immunization with pgH/pgL, pgH/pgO or pgL/pgO could not provide full protection. Co-vaccination with gH, gL and gO pDNAs elicited robust neutralizing antibody and cellular immune responses. Moreover, full protection was also achieved by simply passive immunization with anti-gH/gL/gO sera. These data demonstrated that gCIII complex antigens had fine immunogenicity and might be a promising candidate for the development of HCMV vaccines.

## Introduction

Human cytomegalovirus (HCMV), a beta herpesvirus, is a ubiquitous large enveloped virus that infects 50 to 100% of the adult population worldwide [1]. Although generally asymptomatic in immunocompetent hosts, HCMV infection is a major cause of morbidity and mortality in immunocompromised persons, such as infants following congenital or neonatal infections, transplant recipients, or AIDS patients [2]. HCMV is the leading viral cause of neurodevelopmental abnormality and other birth defects in children and the costs to society are substantial [3,4]. Although antiviral therapy is available [5], the treatment with antiviral agents is imperfect and development of a CMV vaccine is the most promising strategy for preventing CMV infection [6]. Both the health and economic benefits of effective HCMV vaccines could be significant, so the US Institute of Medicine and US National Vaccine Program Office has categorized development of a CMV vaccine as a level 1 (highest-level) priority [7,8], but no candidate vaccine is yet under consideration for licensure.

HCMV envelopment is very complicated and comprises more than 20 glycoproteins which may be the reason for broad cellular tropism of HCMV. Viral envelope contains three major glycoprotein complexes [9]. The gCI complex is comprised of dimeric molecules of glycoprotein B. The gCII complex is a heterodimer consisted of gM and gN protein. The gH, gL and gO together form a unique, high molecular weight gCIII complex. Three constituents are covalently linked by disulfide bonds. These glycoprotein complexes play the crucial role in viral attachment, binding, fusion and entry into the host cell. To develop CMV vaccine blocking virus entry, these glycoproteins are primary target antigens.

Currently the main target for HCMV vaccine development is gB, but clinical studies have demonstrated that gB protein could only offer ~50% protection [10]. For gCII complex, Shen *et al*. reported that HCMV gM and gN DNA vaccines could elicit neutralizing antibody (Nab) response against multiple strains of HCMV [11]. We have also found that immunizing mice with a high dose of MCMV gM/gN DNA vaccine three times could provide mice full protection against a lethal MCMV infection [12]. Fouts *et al*. analyzed anti-CMV hyperimmuneglobulin (Cytogam) and found that neutralizing antibodies in sera from natural HCMV infections mainly targeted the protein complexes consisting of gH-gL, i.e., the heterologous pentamer gH/gL/UL128/UL130/UL131 and the gH/gL/gO complex, instead of the gB protein [13]. This suggests that the gH-gL complexes are the primary targets of the host’s neutralizing antibody response against HCMV infection. Therefore immunogenicity study of gH-gL complexes is necessary. Wussow *et al*. found that immunizing rhesus monkeys with a recombinant poxvirus expressing CMV gH/gL/UL128/UL130/UL131 pentamer could elicit neutralizing antibodies and provide some protection against RhCMV infection [14]. Loomis *et al*. reported that immunizing mice with alphavirus replicon particle (VRP) expressing HCMV gH/gL could induce a robust complement-independent neutralizing antibody response [15]. But so far immunogenicity and *in vivo* protection offered by the whole gCIII complex (gH/gL/gO) have not been reported.

Because of the strict species specificity of CMV infection, there is no animal model available for study of HCMV infection and immunity. Murine cytomegalovirus (MCMV) infection is the most widely used mouse model simulating HCMV infection [16,17]. In the current study, we investigated the immunogenicity and protective efficacy of MCMV gCIII antigens delivered in the form of DNA vaccine. The results demonstrated that gH/gL/gO complex had fine immunogenicity and could provide mice complete protection against lethal SG-MCMV challenge.

## Results

### Detection of gH/gL/gO expression *in vitro*

**Fig 1:**
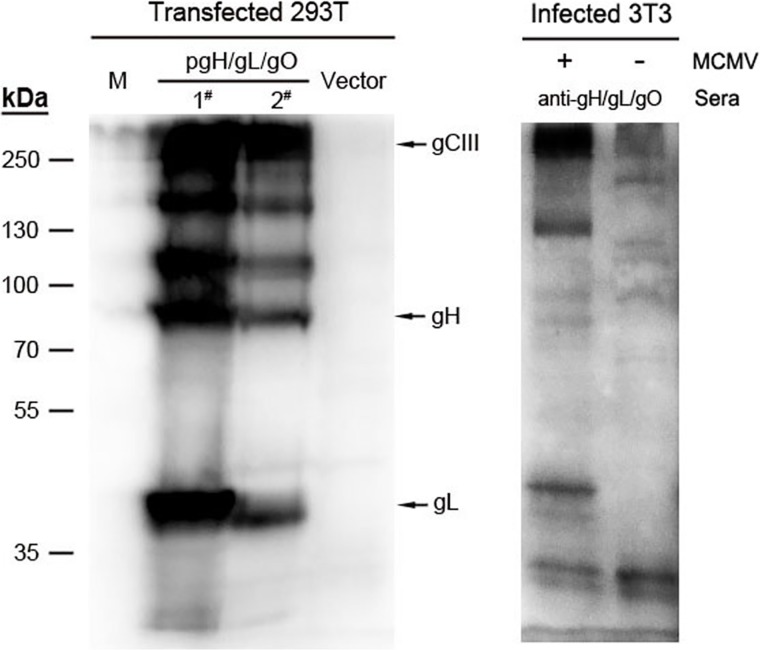
Expression of plasmid DNAs encoding gH, gL and gO of MCMV. 293T cells were transfected with either the vector DNA or co-transfected with gH/gL/gO pDNAs, and lysates were made 48 h later. 3T3 cells were infected with MCMV for 3 days and then lysates were made. Lysate proteins were resolved on a non-reducing SDS-10% PAGE gel and transferred to PVDF membranes and immunoblotted with polyclonal antisera to gH-gL-gO. The lanes labeled as 1^#^ and 2^#^ represented the samples prepared from cells transfected with 2 μg or 1μg each in the mixture of gH/gL/gO pDNAs, respectively.

**Fig 2:**
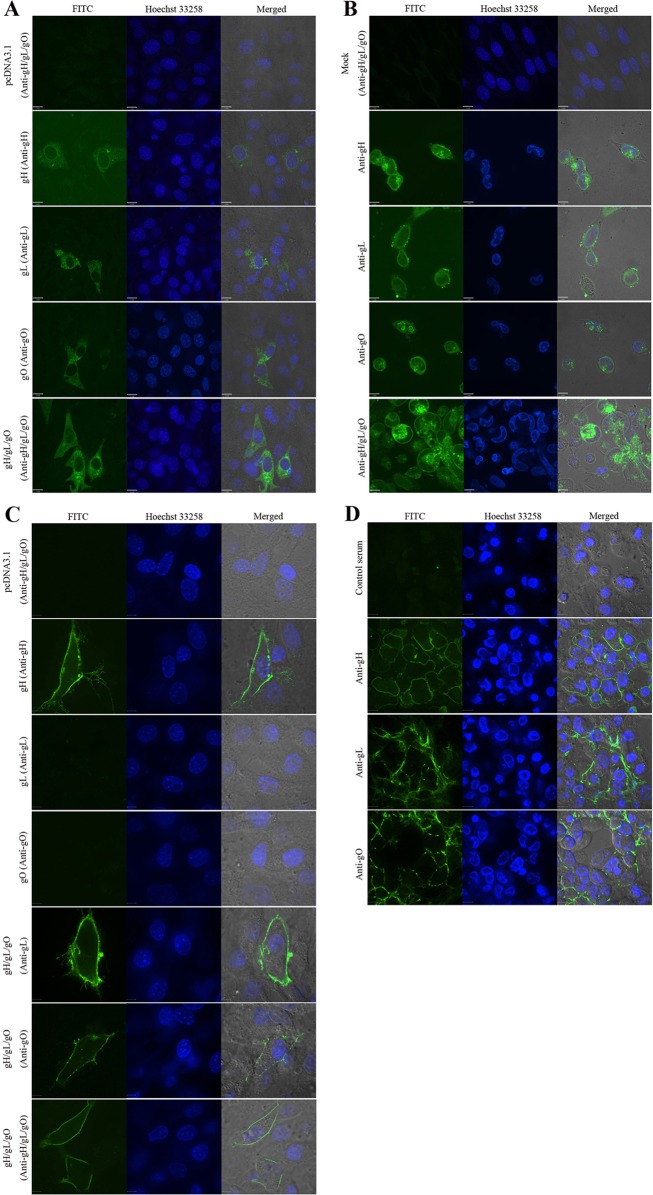
Confocal microscope analyses of pgH, pgL, pgO-transfected (A, C) and MCMV strain Smith-infected 3T3 cells (B, D). 3T3 cells were transfected with empty vector, gH, gL, gO or co-transfected with gH/gL/gO pDNAs and harvested 48 h post-transfection. Strain Smith-infected 3T3 cells were harvested 3 days post-infection. Immunofluorescence assays were performed either with (A, B) or without permeabilization (C, D) to determine the intracellular and surface distribution of three glycoproteins, respectively. Anti-gH/gL/gO antibodies were detected with FITC-conjugated goat anti-mouse IgG (left columns). Nuclei were stained with Hoechst 33258 (middle columns). Bars, 10 μm in A and B, 12 μm in C, and 20 μm in D. Specific immunofluorescence was observed with a confocal laser scanning microscope.

The gH, gL, gO-expressing plasmids were prepared as described in material and methods section. To detect their expression *in vitro*, gH, gL, and gO pDNAs were co-transfected into 293T cells and Western blot was performed. As shown in Fig. 1, three proteins were effectively expressed in the co-transfected cells. The gH protein was ~86 kDa, gL was ~42 kDa, and the ~125 kDa band might be the highly glycosylated mature gO protein, which were consistent with the results of single gene transfection (data not shown). Moreover, a ~250 kDa protein band was detected, which should be the heterotrimer formed by gH, gL and gO proteins, and the size was consistent with the complex detected by Western blot in MCMV infected 3T3 cells. We further performed immunofluorescence assays either with or without permeabilization on pgH/pgL/pgO transfected or MCMV infected 3T3 cells to detect the expression and localization of three proteins. In permeabilized cells (facilitating antibody to enter the cell cytoplasm and nucleus), as shown in Fig. 2A, gH, gL and gO expressed from transfection of a corresponding single plasmid was distributed in the cytoplasm; and gH was also localized on nuclear membrane. When the three proteins were co-expressed, they also mainly localized in the cytoplasm and were not present in the nucleus. For virus infected 3T3 cells (Fig. 2B), three proteins were mainly localized in juxtanuclear regions and were not present in the nucleus, which was consistent with the immunofluorescence observations of HCMV infected cells reported by Theiler *et al* [18]. In addition, staining of nonpermeabilized cells transfected with pgH revealed fluorescent staining was mainly concentrated at the periphery of cells, indicating the presence of cell surface-localized gH protein. Meanwhile, staining of nonpermeabilized cells transfected with single gL or single gO pDNA failed to detect any specific fluorescence. In contrast, when co-transfecting with gH/gL/gO pDNAs, a fraction of gL and gO were detected to be membrane-localized (Fig. 2C). These results indicated that gH is a transmembrane protein and a substantial fraction were readily surface-localized in transfected cells, while gL and gO may display the membrane-localization pattern when interacting with gH by forming the heterotrimer complex (gH/gL/gO). With respect to MCMV infected cells, a substantial fraction of gH, gL and gO were localized in the cell surface (Fig. 2D). Taken together, these results indicated that pgH/pgL/pgO co-transfection yielded effective expression of the proteins, and they shared the same intracellular localization and could form the gCIII complex.

### Protection provided by pgH/pgL/pgO immunization

BALB/c mice were randomly divided into 9 groups. The groups A-D were respectively immunized with 50 μg pgB (positive control), pgH, pgL and pgO; the group I received empty vector DNA (negative control). The groups E, F, G and H were respectively co-immunized with pgH/pgL, pgL/pgO, pgH/pgO and pgH/pgL/pgO at a dose of 50 μg DNA total. Mice were immunized three times at three-week intervals, and were challenged with a lethal dose of SG-MCMV. The protective effects of the DNA vaccines were evaluated comprehensively using infection symptoms, weight loss, residual spleen viral loads and survival.

**Fig 3:**
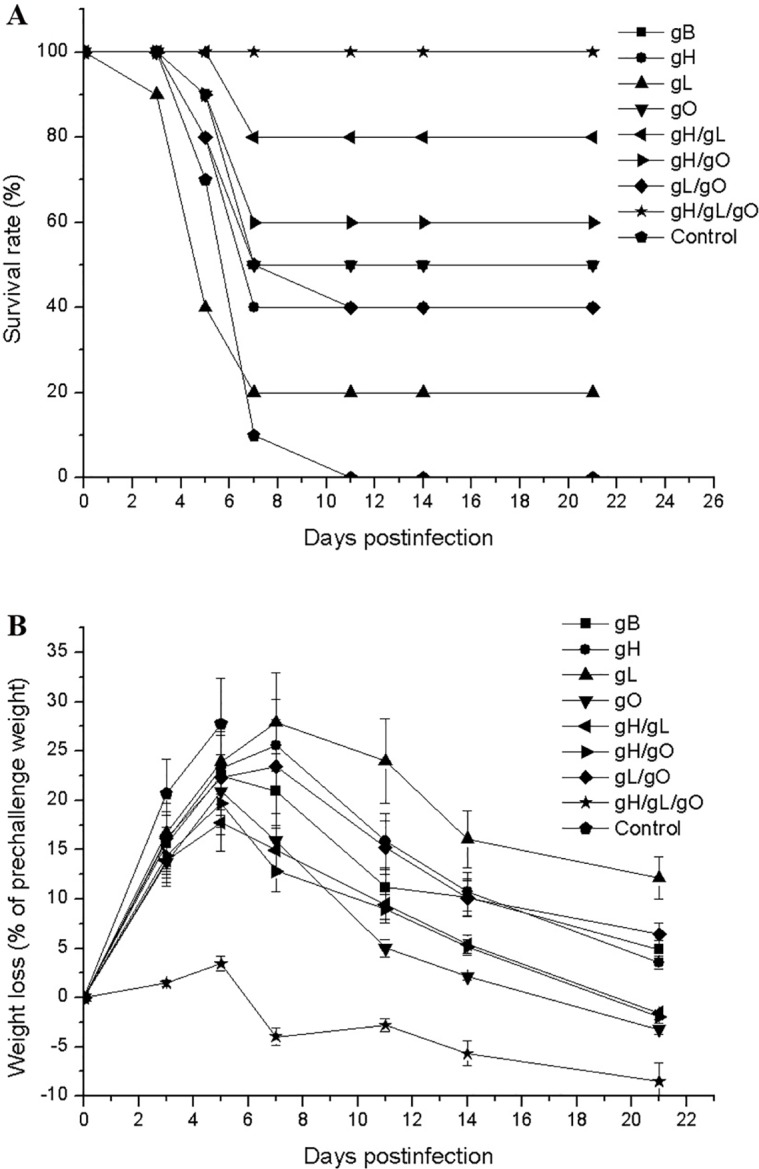
Survival rates (A) and body weight changes (B) after the challenge in the mice immunized with pgH, pgL, pgO or various joint DNA vaccines. Mice were immunized thrice with gB, gH, gL, gO, gH/gL, gH/gO, gL/gO and gH/gL/gO pDNAs at a dosage of 50 μg (25 μg each in the mixture of two DNAs, 16.7 μg each in the mixture of three DNAs). Control mice were immunized with 50 μg vector plasmid. Three weeks after the last immunization, all the mice were challenged with a lethal dose of SG-MCMV. Body weight losses and survival rates of mice were determined 21 days post-challenge. Data points represent mean ± SD in B.

After the lethal challenge, symptoms were the most evident in the negative control group (group I), such as piloerection, lethargy, anorexia, arching back, emaciation, and all mice died within one week of infection. In comparison, the vaccine groups had fewer deaths and no further death occurred after one week (Fig. 3A). For the positive control, the pgB immunized mice had 50% survival. When the four viral antigens were compared for protection against the challenge, there were no significant differences among them. The survival of mice received pgH, pgL, or pgO single DNA immunization was 40%, 20% and 50%, respectively, indicating gH and gO provided similar immune protection as gB while gL was less protective (Table 1). The combination of two genes, gH/gL (Group E), gL/gO (group F), and gH/gO (group G) with 50 μg dose total and three immunizations gave survival of 80%, 40% and 60%, respectively, which showed no significant differences among the two gene co-immunization groups. The best, and the only full protection, was seen in mice given gH/gL/gO three genes co-immunization (group H).

**Table 1:** Protection provided by pgH, pgL, pgO or joint DNA vaccines against lethal SG-MCMV challenge in mice after immunization^a^.

Group	Immunogen	Dose (μg)	Protection against SG-MCMV challenge	
			Spleen virus titers (log_10_ PFU/ml)	Survival mice/tested mice
A	gB	50 μg	5.25±0.16^b^	5/10^b^ ^,^ ^d^
B	gH	50 μg	5.42±0.27^b^	4/10^d^
C	gL	50 μg	5.88±0.32	2/10^d^
D	gO	50 μg	5.35±0.18^b^	5/10^b^ ^,^ ^d^
E	gH/gL	25 μg each	4.75±0.21^b^	8/10^b^
F	gL/gO	25 μg each	5.52±0.32	4/10^d^
G	gH/gO	25 μg each	4.92±0.41^b^	6/10^b^
H	gH/gL/gO	16.7μg each	1.96±0.63^b^	10/10^b^ ^,^ ^c^
I	vector	50 μg	6.18±0.11	0/10

^a^ Mice were immunized with gB, gH, gL, gO, gH/gL, gH/gO, gL/gO or gH/gL/gO pDNAs at a dosage of 50 μg (25 μg each in the mixture of two DNAs, 16.7 μg each in the mixture of three DNAs). Control mice were immunized with 50 μg vector plasmid. Three weeks after the third immunization, all the mice were challenged with a lethal dose (5×LD_50_) of SG-MCMV. Spleen virus titers 5 days after challenge and survival rates of mice 21 days post-infection were determined. Results are expressed as means ± SD of tested mice in each group.

^b^ Significant difference (*p*<0.05), compared with control subjects.

^c^ Significant difference (*p*<0.05), compared with gB immunization group.

^d^ Significant difference (*p*<0.05), compared with gH/gL/gO co-immunization group.

Virus load in the challenged mice was measured by determining spleen virus titer. Very high virus titer was seen in mice in the negative control group, while virus titers were more or less lower in the vaccine groups. The titers in the co-immunization groups were lower than those of the single gene immunization groups. The most remarkable difference was found with the gH/gL/gO co-immunized mice (group H), as its spleen virus titer was about 10^4^-fold lower than the control (group I), indicating that immune responses induced by gH/gL/gO co-immunization could effectively clear virus *in vivo* (Table 1).

Body weight of the challenged mice changed along their health situation. Weight loss was most marked 5–7 days post-infection, for the negative control mice, maximal weight loss reached near 30% (Fig. 3B). The gH, gL, or gO single gene immunized mice had obvious symptoms and weight losses after challenge. Mice in the higher survival groups had less weight losses. In particular, mice given gH/gL/gO co-immunization (group H) showed little signs of infection and their weight losses were significantly less than other immunized groups (Fig. 3B). The above results demonstrated that immunization with a single gH or gO DNA vaccine could offer mice some protection, and co-immunization with all three genes of gCIII (gH/gL/gO) markedly improved the protective efficacy and provided mice full protection.

### Neutralizing antibody response to pgH/pgL/pgO immunization

**Fig 4:**
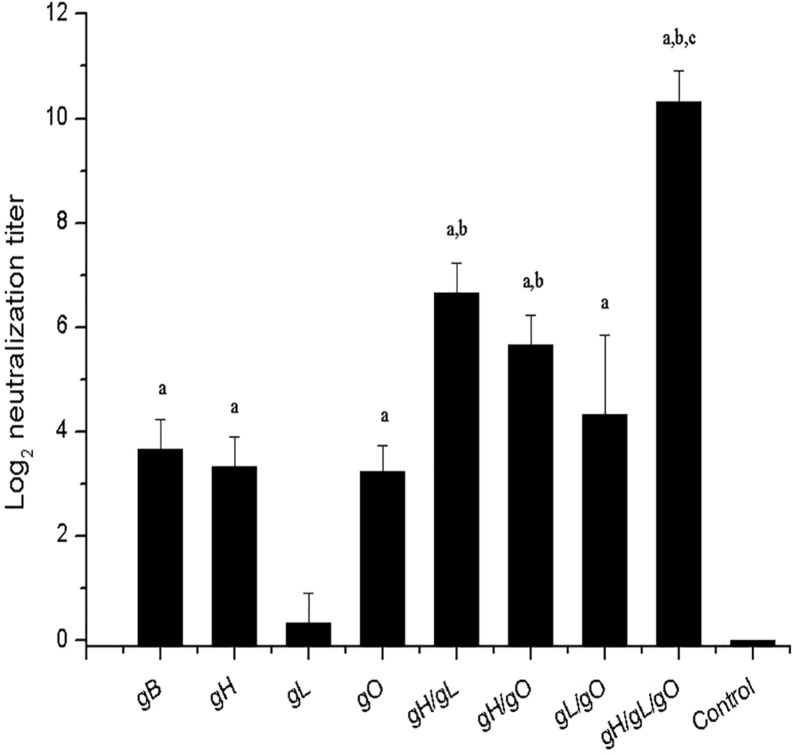
Neutralization titers of mice sera against MCMV. Mice were immunized thrice with gB, gH, gL, gO, gH/gL, gH/gO, gL/gO and gH/gL/gO pDNAs at a dosage of 50 μg (25 μg each in the mixture of two DNAs, 16.7 μg each in the mixture of three DNAs), respectively. Control mice were immunized with 50 μg vector plasmid. Sera were collected 3 weeks after the last immunization. Serum samples were diluted twofold serially. Neutralization titers shown were the highest sera dilutions at which 50% reduction of MCMV infection was achieved. Values represent the geometrical means ± SD of each group. ^a^ Significant difference compared to the mice in control group (*p* < 0.05). ^b^ Significant difference compared to the mice in gH, gL or gO single immunization groups (*p* < 0.05). ^c^ Significant difference compared to the mice in two gene co-immunization groups (*p* < 0.05).

Neutralizing antibodies (Nab) in sera of immunized mice were detected by plaque reduction assay. As shown in Fig. 4, NAb titer was undetectable in the negative control group. The NAb titer levels in the pgB, pgH, pgO single gene immunization groups were similar and the level in the gL group was slightly lower. The NAb levels in the co-immunization groups were higher than those in the single immunization groups. At the immunization dose of 50 μg, the NAb level in gH/gL/gO co-immunization group (group H) was 100+ fold higher than that in the gH single immunization (group B) and the difference was very significant. Also NAb titer in the gH/gL/gO co-immunization group was significantly higher than those from the two gene co-immunization groups. Therefore, co-immunization with gH/gL/gO could induce the best neutralizing antibody responses.

### Cell-mediated immune response to immunization

Cell-mediated immune (CMI) responses to pgH/pgL/pgO immunizations were assessed by measuring IFN-γ secretion in mouse splenocytes. BALB/c mice were immunized with gH, gL, gO or a mixture of three pDNAs at a dosage of 50 μg for three times. Data were presented as the average number of spots in triplicate stimulant wells.

**Fig 5:**
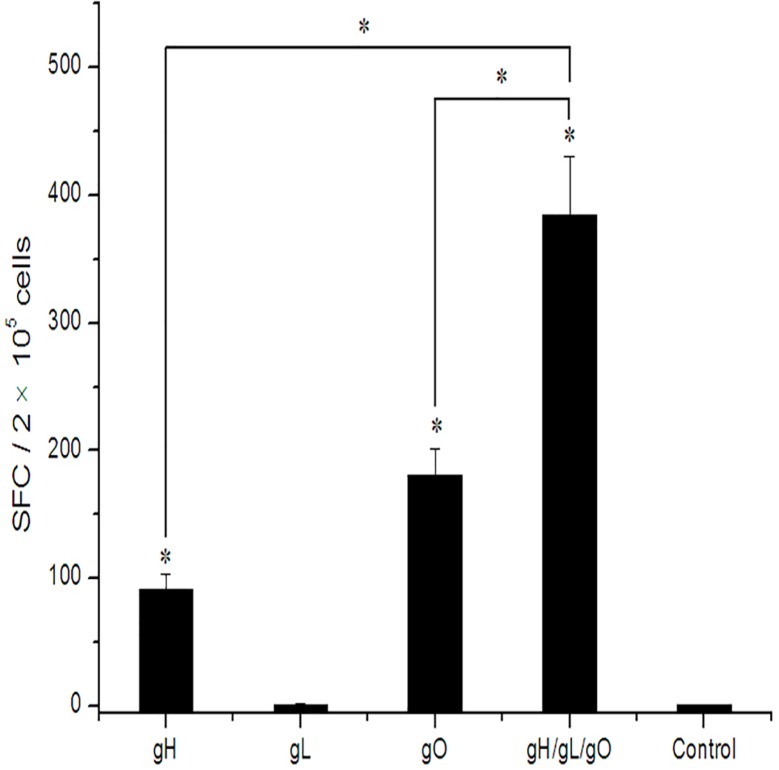
Cellular immune responses of mice vaccinated with gH/gL/gO pDNAs by ELISPOT assay. Mice were immunized with gH, gL, gO alone or a mixture of gH/gL/gO pDNAs at a dosage of 50 μg. Control group was inoculated with vector plasmid. Splenocytes were isolated 2 weeks later and stimulated *in vitro* with 10 μg/ml of the corresponding peptides. The numbers of IFN-γ secreting splenocytes were shown. Values represent the geometrical means ± SD of each group. ^a^ Significant difference compared to the mice in control group (*p* < 0.05). ^b^ Significant difference compared to the mice in gH, gL or gO single immunization groups (*p* < 0.05).

As indicated in Fig. 5, the number of specific IFN-γ secreting splenocytes in the gH, gO and gH/gL/gO immunized mice was significantly higher than that of the control group (*p*<0.05), while no specific response was detected with the two stimulating peptides for the gL immunization group. Also CMI responses in the gH/gL/gO co-immunization group was significantly higher than that in the single gene immunization groups. For the control group, only a small number of non-specific spots were detected by polypeptides (number of spots ≤ 10/10^6^ cells), which was on the same level as the background value of the Elispot plate (un-stimulated splenocytes). The number of positive spots obtained with concanavalin stimulation was as high as 2000/10^6^ cells (data not shown). The above results demonstrated that gH and gO DNA vaccine could elicit a certain level of CMI response and gH/gL/gO co-immunization could elicit a highly effective CMI response.

### Dose-dependent protective efficacy of pgH/pgL/pgO co-immunization

As described above, pgH/pgL/pgO co-immunization could elicit excellent immune protection. To determine the minimum dose of pgH/pgL/pgO for providing mice full protection, mice were immunized with one of the three doses, 50 μg (16.7 μg each), 10 μg (3.3 μg each) and 3 μg (1 μg each) for two or three times, respectively, and then challenged with a lethal dose of SG-MCMV.

As seen from Table 2, the best protection was seen in mice immunized with pgH/pgL/pgO at the 50 μg dose. Twice immunization could already offer 100% protection. For the 10 μg dose group, three immunizations also provided mice full protection, and two immunizations provided 80% protection. The protection was lower in the 3 μg dose group in which three immunizations could provide mice 50% protection. The neutralizing antibody level in the pgH/pgL/pgO co-immunization groups increased as dose and frequency of immunization. Both the 50 and 10 μg dose groups had significantly higher neutralizing antibody level compared with the corresponding 3 μg dose groups. Spleen virus titers showed a clear decreasing trend as immunization dose and frequency increased. The spleen virus titers in all but the 3 μg dose immunization groups were significantly lower than that in the negative control group. Meanwhile, the 50 and 10 μg dose groups had significantly lower virus loads than that in the 3 μg dose groups. Three immunizations of the gH, gL or gO single gene immunization at 50 μg dose provided maximal of 50% protection (Table 1), whereas three gene co-immunization at 3.3 μg each provided 100% protection (Table 2), suggesting that pgH/pgL/pgO co-immunization could induced more stronger immune protection.

**Table 2:** Protection and antibody responses in mice immunized with various doses of gH/gL/gO pDNAs vaccine^a^.

Immunogen	Dosage(μg)	Immunization times	NAb responses (log_2_ neutralization titer)	Protection against SG-MCMV challenge	
				Spleen virus titers (log_10_ PFU/ml)	Survival mice/tested mice
gH/gL/gO	3 (1 μg each)	2	3.67±1.15	5.73±0.19	3/10
		3	5.33±0.58	5.32±0.31	5/10^b^
	10 (3.3 μg each)	2	7.33±0.58^c^	4.81±0.28^b^ ^,^ ^c^	8/10 ^b^
		3	8.00±1.00^c^	4.34±0.41^b^ ^,^ ^c^	10/10^b^ ^,^ ^c^
	50 (16.7 μg each)	2	9.33±1.15^c^	3.39±0.76^b^ ^,^ ^c^	10/10^b^ ^,^ ^c^
		3	10.67±0.58^c^	1.73±0.70^b^ ^,^ ^c^	10/10^b^ ^,^ ^c^
vector	50	3	Undetected	6.05±0.27	0/10

^a^ The mice were immunized twice/thrice, 3 weeks apart, with various doses of gH/gL/gO pDNAs vaccine. Control mice were immunized with 50 μg vector plasmid. Sera were collected 3 weeks after the last immunization. Neutralization titers shown were the highest sera dilutions at which 50% reduction of MCMV infection was achieved. Three weeks after immunization, all the mice were challenged with a lethal dose of SG-MCMV. Spleen virus titers 5 days after challenge and survival rates of mice 21days post-infection were determined. Results are expressed as means ± SD of tested mice in each group.

^b^ Significant difference (*p*<0.05), compared with control subjects.

^c^ Significant difference (*p*<0.05), compared with 3 μg dose groups (analyzing in accordance with the corresponding immunization times).

### Passive immunization with anti-gH/gL/gO sera

As antisera from pgH/pgL/pgO co-immunized mice showed excellent neutralizing activity *in vitro*, passive immunization was carried out to test whether anti-gH/gL/gO sera could protect mice from lethal MCMV infection. Antisera were prepared from mice immunized three times with 50 μg pgH/pgL/pgO DNA vaccine, and the control sera were prepared from mice immunized with the empty vector. The two types of sera were administered to 10 naïve mice via tail vein injection at 300 μl per mouse. Within 24 hours, these mice were challenged with a lethal dose of SG-MCMV. As shown in Table 3, all the mice in the positive control group (pgH/pgL/pgO immunization) survived the lethal challenge and showed no significant signs of infection; all the mice received with the anti-gH/gL/gO sera also obtained 100% protection and only showed slight signs of infection. In contrast, mice received with the control sera were all died. This result demonstrated that the antibody response elicited by pgH/pgL/pgO co-immunization plays important roles in immune protection. This experiment also indicated that passive immunization with anti-gH/gL/gO sera could be used to prevent and treat CMV infection.

**Table 3:** Protection offered by passive immunization with anti-gH/gL/gO antisera^a^.

Immunogen	Protection against SG-MCMV challenge	
	Survival mice/tested mice	Body weight loss (% of the original)
gH/gL/gO pDNAs	10/10^b^	5.19±1.03^b^
Anti-gH/gL/gO sera	10/10^b^	7.62±1.17^b^
Control sera	0/10	28.5±4.16

^a^ Anti-gH/gL/gO serum was collected and pooled from mice immunized thrice with gH/gL/gO pDNAs at a dosage of 50μg (16.7μg each in the mixture of three DNAs). Naive BALB/c mice were passively immunized with the pooled serum by tail vein injection in a volume of 300 μl. Control mice received a corresponding quantity of serum from the mice immunized with vector plasmid. The mice were then challenged with a lethal dose (5 × LD_50_) of SG-MCMV after 24 hours. Body weight losses and survival rates of mice were determined 5 days and 21days post-challenge, respectively.

^b^ Significant difference (*p* < 0.05), compared with control subjects.

## Discussion

Both cellular and humoral immune responses play important roles in the host’s defense against HCMV infections, thus an ideal CMV vaccine should induce both CMV-specific cellular and humoral immune responses [19,20]. The humoral immunity could produce antibody responses against over 100 structural and non-structural proteins of CMV, but the neutralizing antibody response is mainly against virus envelop glycoproteins [21,22]. Neutralizing antibody can prevent entry of cell-free virus which is an important component of adaptive immunity [22]. Therefore, envelop glycoproteins have become the main target antigens for CMV vaccine development. The high abundance glycoproteins in the CMV envelop include gB (gCI), gM/gN (gCII), and gH/gL/gO (gCIII) complex, all of which are necessary for virus replication and infection. All known herpes viruses encode a gH-gL heterodimer to mediate fusion of virus envelop with cell plasma membrane [23,24], indicating the gH-gL complex plays an irreplaceable critical function in the cell infection process of herpes viruses [25]. Antibodies against HCMV gH do not affect virus attachment to cells but could block virus entry into cells and virus spread among cells [26]. Viral mutants with gH-deletion could assemble normally but the progeny viruses could not infect cells, and gL-deleted mutants are also defective in cell entry [27]. For gO deleted mutants, virus titer in infected cells was over 1000 fold lower than the wild type, and the progeny viruses could no longer infect epithelial cells, endothelial cells and fibroblasts [28]. Currently the specific cellular receptor for gCIII complex is not yet clear. It has been reported that gH interact with integrin α_v_β_3_ [29]. Since gCIII complex plays important roles in mediating virus infection and replication, and is a main target of protective immune response after host infection of CMV [30], the current study selected gCIII as vaccine candidate antigen and studied its immune protection efficacy.

Co-transfection of gH/gL/gO pDNAs into 293T cells found that three proteins were effectively expressed, and gH, gL and gO could form gCIII complex. We performed immunofluorescence assays either with or without permeabilization on pgH/pgL/pgO transfected or MCMV infected 3T3 cells to detect the expression and localization of three proteins. In the permeabilized transfected cells, the three proteins were localized in the cytoplasm and not present in the nucleus. In comparison, gH, gL and gO localization in virus infected 3T3 cells showed somewhat different features and were mainly in juxanuclear region, which was consistent with the immunofluorescence observations of HCMV infected cells reported by Theiler *et al* [18]. We infer that in infected cells, gH, gL, and gO proteins mainly localize in cytoplasmic virus assembly compartment (cVAC or AC), which mainly distribute in the juxanuclear region [31]. Tegument proteins and envelope proteins including gM/gN, gB and gH/gL/gO are gathered in the cVAC to facilitate virus enveloping and maturation [31]. In addition, staining of nonpermeabilized cells transfected with gH revealed fluorescent staining was mainly concentrated at the periphery of cells, indicating that gH is a transmembrane protein and a substantial fraction were readily surface-localized in transfected cells, while gL and gO may display the membrane-localization pattern when interacting with gH by forming the heterotrimer complex (gH/gL/gO). In brief, the above experiments demonstrated that the gH/gL/gO pDNAs could be expressed *in vitro* and gH, gL and gO proteins could form a complex; and the three proteins and their complex could be used as antigens for eliciting anti-CMV protective immunity *in vivo*.

Firstly we compared immune protection induced by DNA vaccines of gH, gL, and gO with that of gB. Three immunizations with 50 μg gB, gH, gL, or gO DNA vaccine protected 50%, 40%, 20%, and 50% mice from a lethal MCMV challenge, respectively, indicating that gH and gO are comparable with gB for providing immune protection while gL is less protective. Loomis *et al*. also found that the immunogenicity of HCMV gL is lower than gH, and no neutralizing antibody could be detected from gL immunized mouse sera [15]. However, as gL is the chaperon molecule of gH protein and could promote post-translational modification, folding and intracellular transport of gH [27], we chose to include gL in immunization. In combined immunizations, when the total DNA dose was controlled at 50 μg, the best immune protection was found to be gH/gL/gO co-immunization, which could offer mice full protection and the immunized mice almost showed no signs of infection. Combination of any two of the three genes could not yet provide full protection, while the gH/gL immunization group showed relatively better protection. When the dose for pgH/pgL/pgO co-immunization was decreased to 10 μg (3.3 μg each gene), three immunizations could still provide mice full protection and two immunizations could provide 80% protection. The contrast that with three immunizations of single gH, gL or gO gene at 50 μg could provide 50% protection at most while co-immunization of pgH/pgL/pgO at 3.3 μg each could provide 100% protection indicated that co-delivery of pgH/pgL/pgO enabled substantial improvement in immune protection elicited. As to the reason for excellent synergetic effect elicited by gH/gL/gO co-delivery, one might be three proteins provide more B and T cell epitopes and thus induce stronger immune response; in addition, co-expression of gH/gL/gO might promote post-translational modification and intracellular transport of the corresponding proteins; Moreover, the complex formed by gH/gL/gO might have some new configuration epitopes which are not present in any single component protein. This is supported by a study that some CMV-specific neutralizing antibodies target the epitopes of complexes rather than single component epitopes [15]. It is also noteworthy that the cell surface-localized gCIII complex may play a role in inducing a strong immune response. In short, more diversified epitopes could induce diversified humoral and cellular immune responses, resulting in better immune protections. When gCIII and gCII antigens were compared for immunogenicity and protection efficacy against MCMV challenge, gCIII antigens showed obvious superior results relative to gCII. Our previous work found that immunizing mice with gCII (gM/gN) DNA vaccine requiring a higher dose (100 μg) and at least three times could provide mice full protection [12], while co-delivery of pgH/pgL/pgO at 10 μg could offer mice 100% protection.

The ability to induce neutralizing antibody is an important objective in CMV vaccine development. Envelop glycoproteins has naturally become the key target antigens for inducing neutralizing antibody. In this study, neutralizing antibodies were induced by gH and gO pDNA immunization, but Nab titer in co-immunization groups, in particular, the pgH/pgL/pgO co-immunization group, was significantly higher than that induced by single gene immunization. Under the same total dose, the neutralizing antibody titer in the pgH/pgL/pgO co-immunization group was over 100 fold higher than that in the gH single immunization group. Additionally, antisera passive transfer experiment found that passive immunization with anti-gH/gL/gO sera also offered mice 100% protection against a lethal MCMV challenge and mice showed only slight signs of infections. The experiment well demonstrated that anti-gH/gL/gO sera had potent anti-CMV activity, and this might open a new avenue for using antibodies to prevent and treat CMV infections. Currently in the clinic, intravenous drips of CMV hyperimmunoglobulin biologics Cytogam (CSL Behring) or Cyotect (Biotest AG) are used to treat CMV infections [32]. Cytogam has been demonstrated to be safe and beneficial in pregnant women and transplant recipients [33]. However, these blood products are polyclonal antibodies prepared from CMV sero-positive donors and thus have some unavoidable safety risks. The safety risks could be greatly lowered if antisera could be elicited by one or several antigens of the virus rather than isolated from CMV sero-positive hosts.

Cell-mediated immune (CMI) response plays important roles in the fight against CMV infection, so stimulating CMI is an important requirement for developing a highly effective CMV vaccine. The current study demonstrated that immunization with gH, gO or gH/gL/gO pDNAs could elicit significant CMI response while gL was poor in inducing CMI. In particular, the gO protein was found to be strong in inducing CMI response, which has not been reported before, and this could be partially responsible for its good immunogenicity. The number of IFN-γ secreting splenocytes in the gH/gL/gO co-immunization group was significantly higher than that in the single gene immunization groups, indicating that gH/gL/gO pDNA co-immunization could elicit highly effective CMI response.

A cost-effectiveness study had been performed about CMV vaccine immunization of adolescent females and it argued that CMV vaccination would be cost effective if it could protect their future children against congenital CMV infections, and it also pointed out that a CMV vaccine must reach a protection of over 60% to be effective [34]. The animal experiments in the current study demonstrated that either gH/gL/gO DNA vaccine co-immunization or anti-gH/gL/gO sera passive transfer could provide mice excellent protection. As CMV has complicated latency characteristics and immune escape mechanisms, it is likely difficult to achieve CMV eradication entirely with vaccine immunization. Nevertheless, many evidence demonstrated that even if a measure could not block CMV infection, by lowering virus load in infected individuals it could still offer significant therapeutic benefits for HCMV patients [35]. CMV vaccine is a most pragmatic way to realize this goal. In the currently study, mouse spleen virus titers in all vaccine immunized groups were lower than that in the negative control group. In particular, the spleen virus titer in the pgH/pgL/pgO 50 μg three immunization group was four orders of magnitude lower than that in the negative control mice; the difference was very significant, although it has not yet completely blocked virus infection. Certainly, we need to consider that the virus used for challenge had enhanced virulence via serial passage of salivary gland homogenates and was much more virulent than wild type virus. The challenge method used was a lethal high dose infection, and thus the challenge was far more hazardous than what would occur naturally. Therefore, it is entirely possible that the CMV vaccine presented in this study could elicit better immune protection against naturally occurring common CMV infections.

In summary, our study demonstrated that co-delivery of gH, gL and gO DNA vaccine was more effective in eliciting neutralizing antibody responses and cellular immune responses than that in the single gene immunization groups. Co-immunization with gH/gL/gO pDNAs or passive immunization with anti-gH/gL/gO sera could provide mice complete protection against lethal SG-MCMV challenge. Therefore, these data highlight that gH/gL/gO complex has good immunogenicity and could be included in future HCMV vaccine development.

## Materials and Methods

### Ethics statement

Six- to eight-week-old female BALB/c mice were purchased from the Center for Disease Control and Prevention in Hubei Province, China. They were bred in specific-pathogen-free conditions in the Animal Resource Center at the Wuhan Institute of Virology, Chinese Academy of Sciences. According to the experiment procedures, mice were housed in individually ventilated cages with free access to food and water in a temperature-controlled and humidity-controlled room maintained under filtered positive-pressure ventilation under a 12 hour light/12 hour dark cycle. All experiments involving animals have been reviewed and approved by the Animal Care Committee of Wuhan Institute of Virology (Permit Number: WIVA04201201), in accordance with the animal ethics guidelines of the Chinese National Health and Medical Research Council (NHMRC). All efforts were made to minimize animal suffering.

### Virus and cells

MCMV Smith strain was used and propagated in NIH 3T3 cells. 3T3 and 293T cells were cultured in Minimal Essential Medium (MEM) containing 10% fetal calf serum (FCS). High virulent salivary gland-derived MCMV (SG-MCMV), propagated in BALB/c mice by 10 serial *in vivo* passages for virulence enhancement, was used in challenge experiments. Challenge was performed with 5 × LD_50_ virus stock.

### Plasmid construction and peptides

DNA vaccines were constructed by cloning the complete open reading frame of gH (M75), gL (M115) and gO (M74) gene from MCMV Smith strain into the eukaryotic expression vector pcDNA3.1. The corresponding primers for cloning gH, gL or gO were as follows:

- gH-F: 5'-CGGGATCCATGAAGTTGTCATTAATACTCTCCATCG-3',
- gH-R: 5'-GCTCTAGATTATCTTTTTTGCCGGCACAGCCGGTAC-3';
- gL-F: 5'-CGGGATCCATGATGCCTTTATTATTGCTCATACTG-3',
- gL-R: 5'-CGGAATTCACGGTCTCTTTCGTTGATATTGAGGG-3';
- gO-F: 5'-CGGAATTCATGAACCCCTTATTACTCATGTCG-3',
- gO-R: 5'-TTGGGCCCTCA GACACGGCTAAAGGATATTGAG-3'.

We also constructed gB pDNA as a positive control, which only encoded the extracellular domain of gB. All constructs were verified by sequencing in full. The plasmids were propagated in Escherichia coli DH5α bacteria and purified using NucleoBond Xtra kit (MACHEREY-NAGEL GmbH & Co. KG).

The peptide TGPSVRALME for gH, the peptide VSPPVLSVLV and LHDDGPIRPDPYRF for gL and the peptide TVVGPVNVTTLYK for gO, which were used for IFN-γ Enzyme Linked Immunospot assay (ELISPOT), were synthesized by GenScript Co., Ltd.

### Immunization and challenge

BALB/c mice were vaccinated three times, 3 weeks apart, with empty vector, gB, gH, gL, gO or co-immunized with gH/gL, gH/gO, gL/gO or gH/gL/gO pDNAs at a dosage of 50 μg (25 μg each in the mixture of two pDNAs, 16.7 μg each in the mixture of three pDNAs) by injection into the right quadriceps muscle. Then in vivo electroporation was administered as described by Aihara and Miyazaki [36]. Negative control mice were inoculated with the empty vector pcDNA3.1 by electroporation.

For lethal challenge experiments, the more virulent SG-MCMV was used. The 50% lethal dose (LD50) of SG-MCMV stock was approximately 10^5^ PFU in BALB/c mice. At 3 weeks postimmunization, mice were challenged with a lethal dose (5 × LD_50_, 200 μl / mouse) of SG-MCMV by intraperitoneal injection. This infection could cause systemic virus replication in mice and death of all unvaccinated mice within one week after the challenge. The mice were weighed and checked every day in order to monitor weight loss, apparent physical condition (bristled hair and wounded skin) and behaviour. Then the mice were humanely euthanized via cervical dislocation after chloroform (inhalation excess) in all cases in order to minimize or avoid animal suffering.

### Immunoblotting analysis

293T cells were plated into six-well plates. At 24 h after plating, cells were transfected with vector plasmid or co-transfected with pgH/pgL/pgO using Lipofectamine 2000 (Invitrogen) according to the manufacturer’s instructions. The cells were harvested and lysed 48 h after transfection. For infection study, 3T3 confluent cells were infected with MCMV (m.o.i = 1) for 1.5 h at 37°C. Unadsorbed virus was removed and infection medium (MEM containing 2% FBS) was added. Cells were collected 3 days postinfection and subsequently lysed. Cell lysates were separated using non-reducing SDS-PAGE (10% gel), blotted onto PVDF membranes and immunoblotted with polyclonal antisera to gH/gL/gO. The polyclonal antisera used in the experiment were obtained from immune serum of mice. The anti-gH sera, anti-gL sera and anti-gO sera derived from the corresponding pgH, pgL and pgO immunized mice, respectively. The anti-gH/gL/gO sera were taken from three plasmids pgH/pgL/pgO co-immunized mice.

### Indirect immunofluorescence assay (IIFA)

Immunofluorescence assays were performed in permeabilized and nonpermeabilized cells, respectively. 3T3 cells were seeded on glass coverslips in tissue culture dishes. After 24 h, cells were infected with MCMV at an m.o.i. of 0.1. Cells were collected 3 days postinfection. For transfection, after overnight growth to reach 70% confluence, the 3T3 cells were transfected with gH, gL, gO or co-transfected with gH/gL/gO pDNAs using Lipofectamine 2000 (Invitrogen), and the cells were harvested 48 h later. For permeabilized cell staining, all the cell samples were washed with PBS, fixed with 4% paraformaldehyde, permeabilized with 0.25% Triton X-100, blocked with 5% non-fat milk, and stained with polyclonal antisera to gH, gL or gO. Next, the fluorescein isothiocyanate (FITC)-conjugated anti-mouse IgG (Millipore) secondary antibodies were added and then stained with Hoechst 33258 for 10 min. For nonpermeabilized cell surface staining, unfixed cells were washed and pre-blocked with blocking buffer and then incubated with primary antibody (anti-gH, gL or gO serum) in blocking buffer for 1 hour at room temperature to detect surface-localized viral glycoproteins. After primary antibody incubation and washing, trasfected or infected 3T3 cells were fixed with 4% paraformaldehyde and washed before adding secondary antibody FITC-conjugated anti-mouse IgG. Fluorescent image analysis was performed on a Leica laser scanning confocal microscope.

### Detection of MCMV-specific neutralizing antibody

Mouse serum samples were collected 3 weeks after the last immunization. Serum samples were stored at -20°C until use. Neutralizing antibody directed against MCMV were determined by a plaque reduction assay as described before [37]. Briefly, decomplemented sera (30 μl) were serially diluted 2-fold with MEM. Each dilution was mixed with 100 PFU MCMV in 30 μl of MEM and were then incubated 1 hour at 4°C and 1 hour at 37°C. The mixture was layered onto 3T3 monolayers and PFU were calculated by the standard plaque assay. A neutralization titer was expressed as the highest serum dilution required to achieve a 50% reduction in the number of plaques.

### IFN-γ ELISPOT assay

Mice were immunized with gH, gL, gO, or co-immunized with gH/gL/gO pDNAs thrice at a dosage of 50 μg by electroporation. Two weeks after the third immunization, splenocytes were isolated for ELISPOT assays. According to the instruction manual (U-CyTech, Netherlands), immunospot plates (Millipore, Bedford, MA) were coated with rat anti-mouse IFN-γ mAb, incubated at 4°C overnight and then blocked with 200 μl of blocking solution R. Subsequently, 2×10^5^ lymphocytes were added to the wells in triplicate, stimulated with 10 μg/ml of corresponding gH, gL, gO peptides or gH/gL/gO polypeptides mixture (for co-immunization group). After 18 hours, the lymphocytes were discarded and biotin-labeled anti-mouse IFN-γ Ab antibody was added to each well and incubated at 37°C for 1 h. Next, diluted Streptavidin-HRP conjugate solution was added and incubated at room temperature for 2 hours. Finally, the plates were treated with 100μl of AEC substrate solution and incubated at room temperature for 20 min in the dark. The reaction was stopped by washing with dematerialized water. Spots were quantified by an ELISPOT reader (Bioreader 4000; Bio-sys, Germany).

### Titration of MCMV in spleen

Five days postchallenge, 3 mice from each group were humanely euthanized with chloroform (inhalation excess) and then bled from the heart with a syringe. After bleeding, the spleens was taken out and homogenized in 1:10 (w/v) volume of MEM containing 10% calf serum. The homogenized fluids were centrifuged and the supernatants stored in aliquots at -80°C. Viral loads were determined using a plaque-forming cell assay as described before [12].

### Passive serum transfer and virus challenge

Two groups of 20 mice were immunized thrice with 50 μg gH/gL/gO pDNAs or empty vector pcDNA3.1. Two weeks after the third immunization, 40 mice were humanely euthanized with chloroform (inhalation excess) and blood was obtained by heart puncture. Naive BALB/c mice were passively immunized with the pooled anti-gH/gL/gO sera or control sera by tail vein injection in a volume of 300 μl, respectively. Then 24 hours later, all the mice were challenged with lethal dose (5 × LD_50_) of SG-MCMV. Body weight losses and survival rates of mice were determined 5 days and 21 days post-challenge, respectively. Then, the mice were humanely euthanized via cervical dislocation after chloroform (inhalation excess) in all cases in order to minimize or avoid animal suffering.

### Statistical analyses

The experimental results were evaluated by One-Way ANOVA (SPSS 17.0 software for Windows). The difference was considered statistically significant when *P*-value was less than 0.05. Fisher’s exact test was used to compare survival rates of mice in experimental and control groups.

## Supporting Information

S1 ARRIVE Checklist (DOC)
